# Congenital Deafness Leads to Altered Overt Oculomotor Behaviors

**DOI:** 10.3389/fnins.2020.00273

**Published:** 2020-04-06

**Authors:** Andréanne Sharp, Christine Turgeon, Aaron Paul Johnson, Sebastian Pannasch, François Champoux, Dave Ellemberg

**Affiliations:** ^1^École d’Orthophonie et d’Audiologie, Université de Montréal, Montreal, QC, Canada; ^2^Department of Biomedical Sciences, University of Montreal, Montreal, QC, Canada; ^3^Department of Psychology, Concordia University, Montreal, QC, Canada; ^4^Applied Cognitive Research/Psychology III, Technische Universitaet Dresden, Dresden, Germany; ^5^Department of Kinesiology, University of Montreal, Montreal, QC, Canada

**Keywords:** deafness, oculomotor abilities, brain plasticity, overt eye movement, pursuit task

## Abstract

The human brain is highly cross-modal, and sensory information may affect a wide range of behaviors. In particular, there is evidence that auditory functions are implicated in oculomotor behaviors. Considering this apparent auditory-oculomotor link, one might wonder how the loss of auditory input from birth might have an influence on these motor behaviors. Eye movement tracking enables to extract several components, including saccades and smooth pursuit. One study suggested that deafness can alter saccades processing. Oculomotor behaviors have not been examined further in the deaf. The main goal of this study was to examine smooth pursuit following deafness. A pursuit task paradigm was used in this experiment. Participants were instructed to move their eyes to follow a target as it moved. The target movements have a possibility of four different trajectories (horizontal, vertical, elliptic clockwise, and elliptic counter-clockwise). Results indicate a significant reduction in the ability to track a target in both elliptical conditions showing that more complex motion processing differs in deaf individuals. The data also revealed significantly more saccades per trial in the vertical, anti-clockwise, and, to a lesser extent, the clockwise elliptic condition. This suggests that auditory deprivation from birth leads to altered overt oculomotor behaviors.

## Introduction

It is well established that oculomotor behaviors depend on both visual and vestibular information, but there is also some evidence that auditory functions are implicated in oculomotor behaviors (e.g., Paulsen and Ewertsen, 1966; Rolfs et al., 2005; Valsecchi and Turatto, 2009; Kerzel et al., 2010; Yuval-Greenberg and Deouell, 2011; Zou et al., 2012). For example, it is well-known that rotating a sound around a subject’s head can induce nystagmus (Paulsen and Ewertsen, 1966). Several studies showed that an auditory stimulus can lead to visual saccades toward the source of the sound (Zahn et al., 1978; Zambarbieri et al., 1982; Van Grootel and Van Opstal, 2009) and that the presentation of an auditory stimulus can reduce the rate of saccades (Rolfs et al., 2005; Kerzel et al., 2010; Yuval-Greenberg and Deouell, 2011; Zou et al., 2012). Moreover, one study has shown evidence that gaze position can affect auditory localization accuracy (Maddox et al., 2014) and results from our team suggest that listening or even imagining auditory motion stimulus can induce involuntary eye movements (Landry et al., 2015). Considering the apparent link between auditory input and oculomotor behaviors, one might wonder how the loss of auditory input from birth might influence these motor behaviors.

There are two ways to study oculomotor behaviors. As described by Munoz and Coe (2011), *overt* oculomotor behaviors simply consist of moving the eyes. On the contrary, covert visual selection oculomotor behaviors are more complex. They imply a shift of attention with or without eye movements. All experiments on oculomotor behavior in the deaf have used paradigms that investigate the visual attention aspects of eye movements (e.g., Heimler et al., 2015; Prasad et al., 2015; Jayaraman et al., 2016). Most of these studies demonstrate an increased selective attention for a target in the visual periphery or for a moving target in deaf individuals.

Eye movement tracking enables to extract several components, including saccades and smooth pursuit (Leigh and Zee, 2015). Saccades and smooth pursuit are different and reflect independent processes (saccades: Sparks and Mays, 1990; pursuit: Lisberger et al., 1987). Only one study has measured the ocular performance in the deaf with a classic overt oculomotor behavior task of pro- and anti-saccade (Bottari et al., 2012). Results showed faster saccade latencies and smaller error rates in pro- than anti-saccade trials in deaf, suggesting a possible alteration in the balance between voluntary and reflexive eye-movement. Overt oculomotor behaviors have not been examined further in the deaf. As opposed to saccades, smooth pursuit requires constant regulation by feedback loops (Leigh and Zee, 2015). Smooth pursuit provides a window into processes like movement generation, integrity of combined visual and motor feedback loops and impairment of feedback control (e.g., Robert et al., 2014; Lizak et al., 2016). Here, we aimed to examine smooth pursuit accuracy and saccades in the deaf.

## Methods

### Participants

Twenty-four adults participated in the present study. Twelve were deaf, having a severe to profound hearing loss. Nine of these subjects were congenitally deaf. The three other subjects became deaf in infancy, between 2 and 24 months (one from an unknown illness, one from a hereditary condition and one from a premature birth) (age range 18–42, *M* = 29). All participants had a long-term severe-profound sensorineural hearing loss. When they came for the study they had their more recent audiogram with them. These thresholds were used. All audiogram were done within a year pre-study. All but one participant used bilateral hearing aid and used oral language in addition to lip reading. The only participant to use sign language was the one who do not use hearing aid (see Table 1 for participant characteristics). Twelve control subjects had normal hearing and no otologic problems (age range 18–34, *M* = 26). All subjects had normal or corrected to normal vision. Vision was tested with the Early Treatment Diabetic Retinopathy Study (ETDRS) eye chart at a distance of 1.5 meters. The set criterion was 20/20 for each eye either for normal or corrected to normal vision. All participants had completed a post-secondary education. None of the participants from either group had learning disabilities, neurological problems or other known medical conditions. All participants were consenting volunteers and were treated according to Tri-Council Policy Statement: Ethical Conduct for Research Involving Humans (Medical Research Council of Canada MRRC, 2003). All participants were naïve to the purpose of the experiment.

**TABLE 1:** Participants characteristics.

**Age al test**	**Age of deafness**	**Main Communication mode**	**PTA (R) (dB HL)**	**PTA (L) (dB HL)**	**Hearing Aids**	**Age of first hearing aid use (years)**
21	Birth	Oral	93	55	Bilateral	3
36	Birth	Oral	72	75	Bilateral	24
32	Birth	Oral	85	78	Bilateral	4
18	Birth	Oral	87	85	Bilateral	3
29	Birth	LSQ	113	>113	Ø	Ø
29	2 months	Oral	85	80	Bilateral	2
30	24 months	Oral	78	67	Bilateral	5
27	Birth	Oral	72	70	Bilateral	1–2 months
42	Birth	Oral	58	63	Bilateral	22
25	Birth	Oral	62	82	Bilateral	6
30	Birth	Oral	>105	>105	Bilateral	1
23	3 months	Oral	>98	>98	Bilateral	3

*PTA: Pure tone average (500Hz, 1000Hz, 2000Hz).*

### Eye Movement Recordings

Eye position was acquired non-invasively using a video-based EyeLink 1000 system with a 2000 Hz-upgraded camera (SR Research, Canada). The EyeLink 1000 system records binocular eye position with a sampling rate of 1000 Hz and a spatial resolution of <0.01°. Head movements were restrained by the use of a chin-rest positioned 60 cm from a linearized video monitor (Viewsonic 19″ CRT, 1024 × 768 pixel resolution, 100 Hz refresh rate). A nine-point calibration routine was performed at the beginning of each experimental condition, using the default EyeLink locations. Calibration was repeated if any calibration point was off target by more than 1° or if the average error for all points was above 0.5°. Average calibration accuracy over all participants was 24 min. arc for the central calibration point, and 32 min. arc for the extreme calibration points at the corners of the screen.

Before the beginning of the experiment, proper instructions were presented verbally by the experimenter and displayed on the screen to ensure full understanding of the experimental task by all participants.

Participants were able to clearly understand spoken instructions by using a combination of lip reading and their hearing aids. For the one participant who only communicates using sign language, the instructions were clearly written. The instructions for each condition were presented in writing on the computer screen, allowing all participants to read them. They had as long as they needed to read and understand the instructions, and the experimenter made sure the instructions were understood before the experiment commenced. None of the hearing-impaired participants reported any significant reading difficulty.

### Experiment: Pursuit Task

Participants were asked to track a moving 0.5° circular target. Drift correction was achieved by offsetting the target by 10°, with the location dependent on the type of motion presented.

The drift correction target started in a different location depending on the type of motion. For example, for the target moving up/down, left/right, the initial drift correction was at the center of the screen. For the clockwise/anti-clockwise, the target appeared on the left-hand side of the ellipse.

**FIGURE 1:**
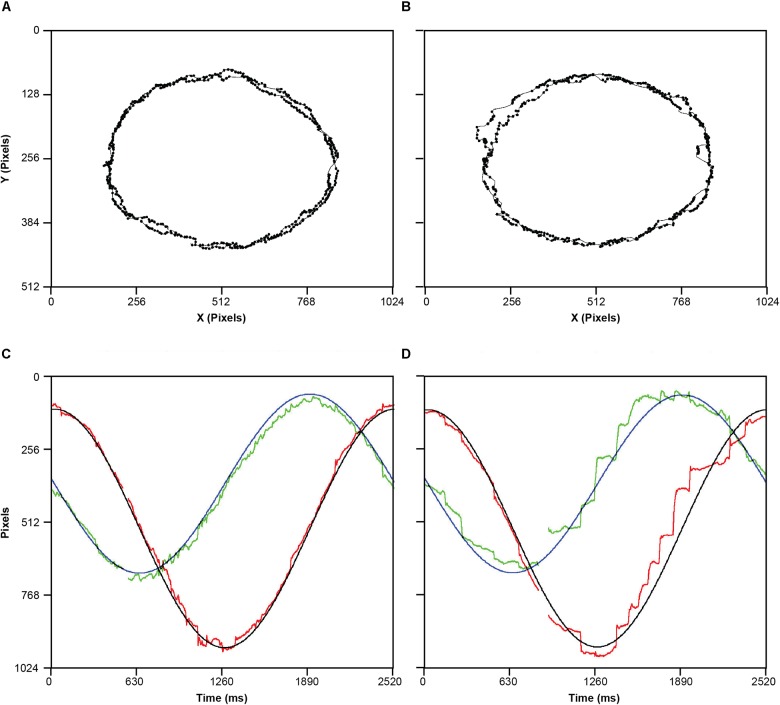
Examples of raw spatial data of eye movements for one control participant **(A)** and one hearing-impaired participant **(B)**. **(C)** a time series representation one cycle of the target elliptical movement in X (Black) and Y (blue) position on the screen, and corresponding tracking eye movements in the X (red) and Y (green). **(D)** Same as C, but for a hearing-impaired participant. Missing data reflect missing samples due to eye blink. Note that all four panels represent the experimental condition where stimulus was moving anti-clockwise at slow speed.

Following the drift correction and a 50 ms blank screen, participants were instructed to move their eyes to follow the target as it moved around the screen in one of four directions: horizontally, vertically, elliptically clockwise, or elliptically counter-clockwise. Moreover, the target moved at one of two velocities (2 or 4 deg/s), resulting in eight conditions repeated three times randomly. Inter-trial interval was self-paced, as the participant had to stare at the fixation marker and press spacebar to initiate the next trial. For an example of raw data for both groups see Figure 1.

The position of the target moved depending on task. For up/down, left/right, the target started at the center of the screen (512,384 in X/Y pixel coordinates). In the up/down condition, the target would then move 5° upward, stop, and then 10° downward, stop, and then finally 5° upward to stop back at the center of the screen. Same is true for the left/right condition.

For the elliptical motion, the target moved in the same direction (clockwise or anti-clockwise) forming a circle with a total radius length of 20°. In all cases, the moving stimulus was presented for 10°s. For both of the elliptical condition, the target started at coordinates on the left of the screen (171,384), with the target proceeding to rotate either clockwise or anticlockwise from that position. The parameters of the ellipse were: Amplitude X axis 341 pixels. Amplitude Y Axis 256 pixels. Target was white (RGB = 255,255,255), background was black (RGB = 0,0,0).

### Data Analysis

Only the data from the right eye were used. Blink periods were identified using the EyeLink 1000 heuristic filtering algorithm (Stampe, 1993) and were removed. In addition, all samples 200 ms before and after each blink were removed to eliminate the initial and final phases of the blink during which the pupil could be partially occluded. We also removed portions of the data that contained very fast increments and decrements in pupil area (20 units per sample). Martinez-Conde et al. (2006) identified these periods as partial blinks that do not fully occlude the pupil and that are not detected by the Eyelink 1000 algorithm. The remaining eye movement data were analyzed after blink and partial-blink data cleanup.

For the pursuit task, accuracy was measured by calculating an individual’s ability to maintain fixation within a 1° radius of the target during the pursuit. This was calculated every 20 samples (i.e., every 20 ms) and percent accuracy was obtained for each trial. The analysis ran using a dynamic region of interest that followed the position of the target on each sample of the eye tracker. Individuals were classified as being accurate if their eye position was within a 1° window around the target during the sample period, or inaccurate if their eye position exceeded the 1° window.

The accuracy (and all subsequent dependent variables) were then entered into a linear mixed effects model. Linear mixed effects models can account for inter-participant and inter-item variation by incorporating random effects in the model design, which is introduced by variation due to individual differences. Their principled methods of modeling heteroskedasticity and non-spherical error variance give linear mixed effects models more power than traditional measures (Baayen et al., 2008). Separate linear mixed effect models on accuracy were completed for each of the four direction (horizontal, vertical, elliptical clockwise, and elliptical counter-clockwise). To predict accuracy, we included one between-subjects factor with two levels (hearing-impaired and controls) and a within-subjects factor of speed with two levels (2 and 4 deg/s) as fixed and random effects—which allowed them to vary across participants. Note we used the Satterwaite method for degrees of freedom. In addition to accuracy, we also calculated mixed effect models with the following dependent variables (1) the number of saccades per trial, (2) saccade latency (time from onset of the stimulus until the first saccade was initiated), (3) saccade amplitude, (4) saccade duration, and (5) average velocity of saccade, for each of the four directions. In addition to the mixed effect models, *post hoc* analysis include Hedges’ *g* as an effect size measure appropriate for small sample sizes, and two-tailed Mann-Whitney tests.

We used a linear model because with large samples sizes, the binomial is well approximated by a Gaussian distribution. To confirm this, we assessed the normality of these data using the Shaprio-Wilk test (all *p* > 0.05 indicating these data were not significantly different from a normal distribution). Further, the linearity of the distribution on a quantile–quantile (Q–Q) plot confirmed these data to be normally distributed. After removing blinks, on average 93% of the sample information was retained for use in the analysis.

## Results

**FIGURE 2:**
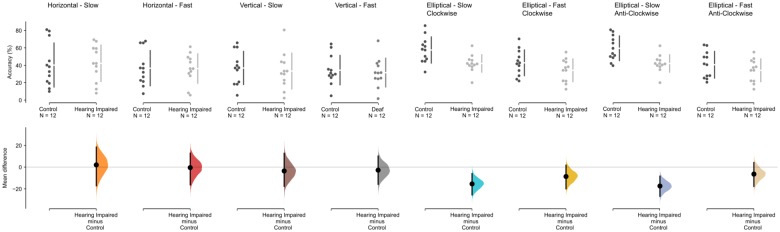
Accuracy results for the pursuit conditions for the hearing-impaired group (light gray) and the control group (dark gray). The mean difference for accuracy (% time on target) for all eight conditions are shown in the above Cumming estimation plot. The raw data is plotted on the upper axes; each mean difference is plotted on the lower axes as a bootstrap sampling distribution (5000 bootstrap samples were taken; the confidence interval is bias-corrected and accelerated). Mean differences are depicted as dots; 95% confidence intervals are indicated by the ends of the vertical error bars.

Figure 2 presents the accuracy measured during the pursuit conditions for the hearing-impaired group (light columns) and the normal hearing control group (dark columns) for each of the four pursuit tasks (vertical, horizontal, elliptic clock-wise, and elliptic anti-clock-wise) at two speeds: slow (2° s^–1^) and fast (4° s^–1^). Accuracy in tracking the target was measured as the percentage of the 10 s trial where the participant-maintained fixation within a 1° radius of the target. The analysis of accuracy in performing the task show significant differences between the groups, *F*(1,33) = 11.082, *p* = 0.002, and between speeds, *F*(1,31.2) = 9.507, *p* = 0.004, but no interaction, *F*(1,33) = 0.888, *p* = 0.353, during the clockwise ellipse task. Likewise, there was a main effect of group, *F*(1,33) = 12.34, *p* = 0.001, and speed, *F*(1,30.7) = 15.28, *p* < 0.001, during the anti-clockwise ellipse task, but no interaction, F(1,33) = 2.61, *p* = 0.116. As seen in Figure 2, there was no effect of group, no effect of speed, and no interaction during the horizontal and vertical pursuit task (all *p* > 0.1). *Post hoc* comparisons show a decrease in mean difference in accuracy between controls and hearing impaired participants for the slow clockwise ellipse (*M*_diff_ = −15.440 [95%CI −25.544, −5.976], Mann Whitney *U* = 118, *p* = *0.009*, Hedges’ *g* = −1.175 [95%CI −1.928, −0.256]) and slow anticlockwise ellipse (*M*_diff_ = −17.382 [95%CI −26.884, −8.3], Mann Whitney *U* = 122, *p* = 0.004, Hedges’ *g* = −1.375 [95%CI −2.137, −0.502]).

**FIGURE 3:**
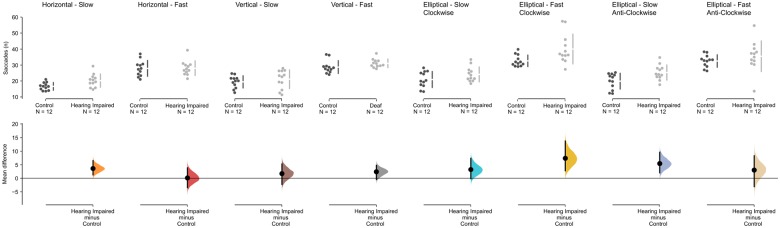
Saccade results for the pursuit conditions for the hearing-impaired group (light gray) and the control group (dark gray). The mean difference for the number of saccades for all eight conditions are shown in the above Cumming estimation plot. The raw data (number of saccades) is plotted on the upper axes; each mean difference is plotted on the lower axes as a bootstrap sampling distribution (5000 bootstrap samples were taken; the confidence interval is bias-corrected and accelerated). Mean differences are depicted as dots; 95% confidence intervals are indicated by the ends of the vertical error bars.

We also analyzed different measurements that affect eye movement during pursuit task, specifically number, duration, latency, amplitude and velocity of saccades. For the average number of saccades (see Figure 3) performed in the anti-clockwise ellipse task, there was a main effect of group *F*(1,32.7) = 6.047, *p* = 0.019, and speed *F*(1,17) = 37.284, *p* < 0.001, but no interaction, *F*(1,33) = 0.505, *p* = *0.482*. There was also a main effect of speed in the clockwise condition *F*(1,32.1) = 65.77, *p* < 0.001, and a main effect of group, *F*(1,28.3) = 8.83, *p* = 0.006. Interestingly (given the results of the task accuracy) there was a main effect of speed, *F*(1,19.2) = 80.662, *p* < 0.001, for the vertical pursuit task, but no main effect of group, *F*(1,11.2) = 1.694, *p* = 0.219. In the horizontal condition, there was a significant main effects of speed, *F*(1,13.7) = 62.02, *p* ≤ 0.001, but not group, *F*(1,5.2) = 2.80, *p* = 0.115. In addition, the analysis did not show any significant interaction (all *p* > 0.1) between group and speed in any of the four pursuit tasks. For all other eye movement measures (average of saccade amplitude, latency until the first saccade, duration and velocity of the saccades), the mixed effect models showed no main effect of group for all conditions (vertical, horizontal, elliptic clock-wise, and elliptic anti-clock-wise: *p* > 0.1).

Focusing on ellipse tracking results, using the *post hoc* analysis we observed that for the fast clockwise ellipse tracking task, there is a significant increase in the number of saccades by the hearing impaired group in comparison to the control group (*M*_diff_ = 7.38 [95% CI 2.762, 13.736], Mann Whitney *U* = 32, *p* = 0.002, Hedge’s *g* = 1.02 [95% CI 222, 1.62). Similarly, we find a similar increase in number of saccades for the hearing impaired group in the slow anti-clockwise condition (*M*_diff_ = 5.435 [95% CI 2.021, 9.6], Mann Whitney *U* = 34, p = 0.003, Hedges’ *g* = 1.09 [95% CI = 0.337, 1.82]).

## Discussion

In the present study, eye movements were tested in a group of hearing-impaired individuals who are deaf since early infancy using a pursuit task. The findings indicate that hearing-impaired participants were less accurate at maintaining target fixation than the control group for the more complex motion in the elliptical tasks. However, hearing-impaired participants performed the same as the controls in the linear motion tasks. The results also indicate that, compared to a control group with normal hearing function, individuals with impaired hearing show a reduced ability to track a target in the elliptical conditions, and made more saccades per trial in the vertical, anti-clockwise, and to a lesser extent, clockwise elliptic conditions at both of the tested speeds. These results suggest that deafness has an impact on the development or maintenance of overt oculomotor behaviors.

Several studies suggest that deprivation of a sensory modality can alter the development of other modalities (Bavelier and Neville, 2002). Cerebral plasticity following deafferentiation can lead to adaptive or maladaptive behavioral changes (Merabet and Pascual-Leone, 2010). In the deaf individual, several studies suggest better performance for a few visual abilities, including visual detection in the periphery (for a summary see Bavelier et al., 2006). However, such specific improvement in performance has not been found in other sensory systems or processes. In regard to the motor system, auditory deprivation appears to lead to maladaptive behavioral changes. Indeed, deafness generally leads to decreased performance on general dynamic coordination, balance, ball catching, reaction times, speed of movement execution and motor learning (e.g., Wiegersma and Velde, 1983; Gayle and Pohlman, 1990; Siegel et al., 1991; Hartman et al., 2011). Our results are in accordance with the general notion that hearing has an important role in the emergence and maintenance of motor processing.

Since eye movements are simpler than other movements in many ways, the oculomotor system provides an ideal opportunity to investigate the brain mechanisms underlying visually guided movement (Lisberger et al., 1987). Nevertheless, despite a relatively large number of studies, most results are contradictory and it is unclear what the effects of auditory deprivation are on visual orienting, for example (for a review see Dye and Bavelier, 2013). Some studies suggest that when visual periphery and central visual field are simultaneously stimulated, deaf individuals are able to further divide more efficiently their visual attention resources in opposition to control participants (Dye et al., 2009). Many experiments showed that deaf individuals are more readily distracted by non-pertinent distracting elements, especially when those elements appear in the peripheral vision field (Proksch and Bavelier, 2002; Chen et al., 2006). All of these studies aimed to investigate complex attentional processes or pursuit of visual stimuli in the periphery and assessed exclusively visual attention orienting, neglecting whether overt oculomotor behavior may also change in deaf people.

Surprisingly, the covert visual selection aspect of visual oculomotor behaviors has been further investigated than the overt aspect. The study of Bottari et al. (2012) is the only one to date to have measured the ocular performance with a classic overt oculomotor behavior task of pro- and anti-saccade. Results of this study suggest a possible alteration in the balance between voluntary and reflexive eye-movement orienting, shorter saccade latencies and smaller error rates were found in pro-saccade trials rather than in anti-saccade trials in both populations. However, the effect was substantially larger in deaf than hearing participants. Our results confirmed those of Bottari et al. (2012), suggesting that movement generation is altered in the deaf, but we extended the latter by suggesting that combined visual and motor feedback loops, and feedback control, which can be assessed with smooth pursuit (Lizak et al., 2016), is also impaired in this population. Taken together, these data suggest that early auditory input is essential for the normal development of the mechanisms underlying the control of eye movements.

The homogeneity of the group present in this study, in terms of severity, age of onset, progression, and etiology should be taken into consideration when interpreting the present findings. All participants had a severe or profound hearing loss (see Table 1 for details). The majority of the participants had a hearing impairment from birth while only three became impaired pre-lingualy, namely between the ages of 2 to 24 months. Finally, all but one participant used hearing aids for amplification. Here, the group of participants all had similar onset of hearing loss, duration of hearing loss, hearing aid use, and modes of communication, factors that have been revealed to critically impact plasticity and behavior in the deaf (e.g., Kral and Sharma, 2012). It is to be noted that the overall variance of their results is quite small and is not different than that of the control group. The many characteristics of hearing loss should be examined further in order to reveal which features trigger more oculomotor behavioral alterations. Future research needs to examine the effect of these characteristics, the kind of behavioral alteration, and whether there exists a critical period during which auditory input is required to develop typical oculomotor behaviors.

Finally, vestibular function may also be discussed in relation to the data. Indeed, a large proportion of congenitally deaf individuals have concomitant vestibular impairment (Buchman et al., 2004). Vestibular cells are involved in vestibule-ocular reflexes, which allow us to hold images still on the retina during brief head movements (Müri and Nyffeler, 2008). In our study, participants had their head fixed, so vestibulo-ocular reflexes were not generated during the experiment. However, neurons from the vestibular nucleus are also involved in signaling eye velocity during smooth pursuit (Katz, 2002; Krauzlis, 2004), so they were involved in the pursuit task. Further studies will need to identify the exact impact of auditory deprivation on oculomotor behavior by controlling for vestibular impairment.

## Data Availability Statement

The datasets generated for this study are available on request to the corresponding author.

## Ethics Statement

The studies involving human participants were reviewed and approved by all participants were consenting volunteers and were treated according to Tri-Council Policy Statement: Ethical Conduct for Research Involving Humans (Medical Research Council of Canada, MRRC, 2003). The patients/participants provided their written informed consent to participate in this study.

## Author Contributions

AS, CT, and DE designed and performed the experiment. All authors wrote the manuscript and discussed the results.

## Conflict of Interest

The authors declare that the research was conducted in the absence of any commercial or financial relationships that could be construed as a potential conflict of interest.
